# Quality indicators of public maternity units in the governorate of Monastir (Tunisia)

**DOI:** 10.1186/s12884-023-05781-5

**Published:** 2023-10-16

**Authors:** Amani Maatouk, Amel Gara, Meriem Kacem, Manel Ben Fredj, Imen Zemni, Hela Abroug, Cyrine Bennasrallah, Wafa Dhouib, Samia Grira Said, Saber Garrach, Ines Zouari, Hayfa Bergaoui, Falah Raja, Ines Bouanene, Asma Belguith Sriha

**Affiliations:** 1 Department of Epidemiology and Preventive Medicine, University Hospital Fattouma Bourguiba of Monastir, Monastir, Tunisia; 2 The Regional Direction of Primary Health of Monastir, Monastir, Tunisia; 3 Department of Obstetric and Gynecology, University Hospital Fattouma Bourguiba of Monastir, Monastir, Tunisia

**Keywords:** Pregnant women, Parturitions, Maternity hospital, Hospital birth center, Tunisia

## Abstract

**Introduction:**

Increasing access to healthcare for expectant mothers is a national goal. In Monastir, Tunisia, some Peripheral Maternity Units (PMUs) required assessment. Our goals were to describe the delivery activities in MUs (maternity units) and to assess whether some of PMUs need to have their activities replaced.

**Method:**

We analyzed aggregate data of deliveries in Monastir from 2015 to 2020. The gouvernorate’s seven public MUs were included. Only the morning activity was allotted for obstetricians and gynecologists, in RMUs 1 and 2, whereas they were not available in all PMUs. Data was gathered from the reports of the National Perinatal Program. Both the availability of Comprehensive Essential Obstetric Care (CEOC) and Basic Essential Obstetric Care (BEOC) were calculated. Trends were calculated using Joinpoint software. The Annual Percent Change (APC) was calculated.

**Results:**

The number of births decreased from 2015 to 2020 (APC= -4.3%: 95%CI : -6; -2.4; p = 0.003). The largest significant decreases in APCs of deliveries were reported in PMU 2 (APC = -12.6% (95%CI : -20; -4.4; p = 0.014), in PMU 3 (APC = -29.3% (95%CI : -36.5; -21.4; p = 0.001), and in PMU 4 (APC = -32.9% (95%CI: -49.1; -11.5); p = 0.016). If PMU 3 and 4 were no longer operating as maternity facilities, BEOC and CEOC standards would still be adequat. For accessibility, both PMU 3 and PMU 2 are accessible from PMU 4 and PMU 1, respectively.

**Conclusions:**

Pregnant women prefer to give birth in obstetric services with ability to perform emergency caesarean at the expense of PMU. Nowadays, it appears that accessibility is less important than the presence of qualified human resources when a pregnant woman choose a maternity hospital.

**Supplementary Information:**

The online version contains supplementary material available at 10.1186/s12884-023-05781-5.

## Introduction

Reproductive health is a major global concern [1]. Improving the availability, access, use and quality of services had made possible to manage complications during pregnancy and childbirth and consequently reduce maternal mortality [2].

The maternal mortality rate in low-income countries in 2020 was 430 per 100 000 live births versus 12 per 100 000 live births in high income countries. Pre-eclampsia and eclampsia, infections, excessive bleeding after delivery and unsafe abortion account for about 75% of all causes of maternal deaths. According to WHO, all women need access to high quality care in pregnancy, and during and after childbirth. Maternal health and newborn health are closely linked. It is particularly important that all births are attended by skilled health professionals, as timely management and treatment can make the difference between life and death for the women as well as for the newborn [3].

Health indicators are useful tools for assessing needs, monitoring and evaluating program implementation and impact. These indicators make it possible to follow the progressive evolutions and the gaps between establishments on a geographical basis [4]. In Tunisia, the National Perinatal Program was set up in 1990. It is made up of nine components of which the third is childbirth in an assisted environment. One of its tools is to improve access to public care for pregnant women. As a result, the health system has set up Maternity Units (MUs) in different districts. Public health projects are based on a balance between needs, demands and supply of health care [5]. Nevertheless, in Monastir (Tunisia), several primary health centers and peripheral maternity units (PMUs) have been created following a request from users and a subsidy from the political authorities of the region. These facilities should be assessed by healthcare facilities and healthcare professionals. During the last three decades, the socio-economic conditions of women has improved in Tunisia leading to a better financial situation. Moreover, a better coverage of medical insurance has led to a better access to the private sector. In fact, since 2004, the National Health Insurance Fund has started to cover deliveries. The infrastructure has improved, making transportation more accessible. Finally, the use of social media to select the highest quality of service has widened the gap in the demand for care. This study was conducted at the Ministry of Health’s request to provide scientific support for the replacement of certain activities of peripheral maternity and removal of childbirth activities in favor of follow-up consultations about pregnancy and family planning.

The objective of our study were to describe the delivery activities in MUs and to assess whether some of PMUs need to have their activities replaced.

## Methods

### Study design

We analyzed aggregate data of deliveries in Monastir from 2015 to 2020, including all the MUs of Monastir governorate.

### Study setting

The governorate of Monastir is a coastal town which is located in the centre-east of Tunisia. Monastir has an area of 1024 km² [6] (0.62% of the total area of Tunisia). The total population of Monastir increased from 561,100 to 2015 to 601,382 in 2018 [7]. In this governorate, a road infrastructure covers the totality of its territory and it has a high disponibility of common transportation (railway transport, common taxi, bus transportation, transport by hire and an international airport).

The governorate of Monastir is composed of 13 delegations. There are 7 public MUs: a University Maternity Unit (UMU), 2 Regional Maternity Units (RMUs) and 4 Peripheral Maternity Units (PMUs). During the morning activity, the obstetrician-gynecologists assigned to RMUs 1 and 2 and additional contractual obstetrician-gynecologists in PMUs 1 and 2 were on hand. During the guards, general practitioners and family medicine residents ensuring emergency services were requested when necessary. However, neither an obstetrician-gynecologist nor an emergency physician was accessible in PMU3 or 4.

### Study population

All deliveries in public MUs of Monastir were included.

### Variables

We analyzed variables to evaluate public maternity units: indicators of use (admission for delivery, number of women giving birth and transfers for delivery), availability of basic essential obstetric care (BEOC), comprehensive essential obstetric care (CEOC) and accessibility of services (infrastructure, geographic and economic accessibility). Geographic and economic accessibility were estimated via Google Maps: distances travelled by car in kilometers (Km), time spent in minutes (min) between maternity units of Monastir [8]. Travel price was estimated in Tunisian Dinar (TND) (1 TND = 0.31 Euros on 21 October 2022 [9]. We also analyzed variables related to outcome indicators: the trends of deliveries according to maternity units and to human resources (deliveries per midwife), maternal mortality, stillbirths and newborns who died in the delivery room. Variables were chosen according to WHO guidelines [10–12].

### Data source and measurement

Reports from the National Perinatal Program were used to compile the data. Weekly health activity reports are collected by Monastir’s Regional Direction of Primary Health; raw data are not available to it.

The total number of births in Monastir per year (in the public and private sector) was acquired from the National Statistical Institute for 2015, 2016 and 2017, and by the Regional Directorate of Primary Health Care for 2018, 2019, and 2020.

Appendix A details the indicators calculated to describe the activity of MUs. Maternal death refers to the death of a woman during pregnancy or during the first 42 days following pregnancy, regardless of the location and duration of the pregnancy, due to any cause related to or complicated by pregnancy or its management, but not due to incidental or accidental causes [1]. Stillbirth is defined as the loss or the death of a baby that occurs prior to or during delivery [13]. For availability indicators, the BEOC is defined as the number of facilities ensuring functioning basic essential obstetric care per 500 000 population [1]. A BEOC facility is that which ensures all of the following 6 services at least once in the previous 3 months: oxytocics and anticonvulsants, administration of parenteral antibiotics, removal of retained products (for example manual vacuum aspiration), manual removal of the placenta, and assisted vaginal delivery (forceps or vacuum extraction). The recommended minimum acceptable level is equal to 4 BEOC facilities per 500 000 population. Moreover, we calculated the CEOC, defined by the number of facilities with functioning CEOC per 500 000 population. A CEOC facility is that which performs surgery (caesarean section) and blood transfusion, as well as all the 6 BEOC services, at least once in the previous 3 months. The recommended minimum acceptable level is equal to 1 CEOC facility per 500 000 population. The mean price of one liter of gasoline in Tunisia during study period was 2.095 TND [14].

### Data analysis

We used the Statistical Package for the Social Sciences (SPSS) version 21.0 to analyze data. We calculated frequencies and percentages for the qualitative variables. Trends were calculated using Joinpoint software. The Annual Percent Change (APC) was calculated. Spearman’s Rho coefficients (r’) were calculated. A p-value less than 0.05 is statistically significant.

## Results

### Description of human resources according to maternity units

During study period, we observed an increase in the number of obstetrician-gynecologists and a decrease in the number of midwives performing primary perinatal activities (r’ = 0.978 (p < 10^− 3^), and r’= − 0.978, (p < 10^− 3^) respectively) (Table 1).

**Table 1:** Distribution of obstetrician-gynecologists and midwives in public sector of the governorate of Monastir from 2015 to 2020 (Tunisia)

Year	Total number of OG	OG performing in PMU and RMU n (%)	Total number of midwives	Midwives performing in PMU and RMU n (%)
**2015**	10	2 (20)	115	34 (29)
**2016**	12	2 (16)	108	32 (29)
**2017**	13	2 (15)	102	31 (30)
**2018**	13	2 (15)	96	30 (31)
**2019**	15	3 (20)	90	28 (31)
**2020**	15	4 (26)	90	28 (31)
B (ES)*	0.951 (0.034)	0.420 (0.066)	-5.340(0.042)	-1.236(0.018)
r’ **	0.963	0.923	-0.978	-0.978
**p-value**	< 10^− 3^	< 10^− 3^	< 10^− 3^	< 10^− 3^

OG: Obstetrician-gynecologist; PMU: Peripheral Maternity Unit; RMU: Regional Maternity Unit; * **B (ES)**: Non-standardized coefficient (standard error); ** r’: Spearman’s Rho

### Description of deliveries according to maternity units

**Fig. 1:**
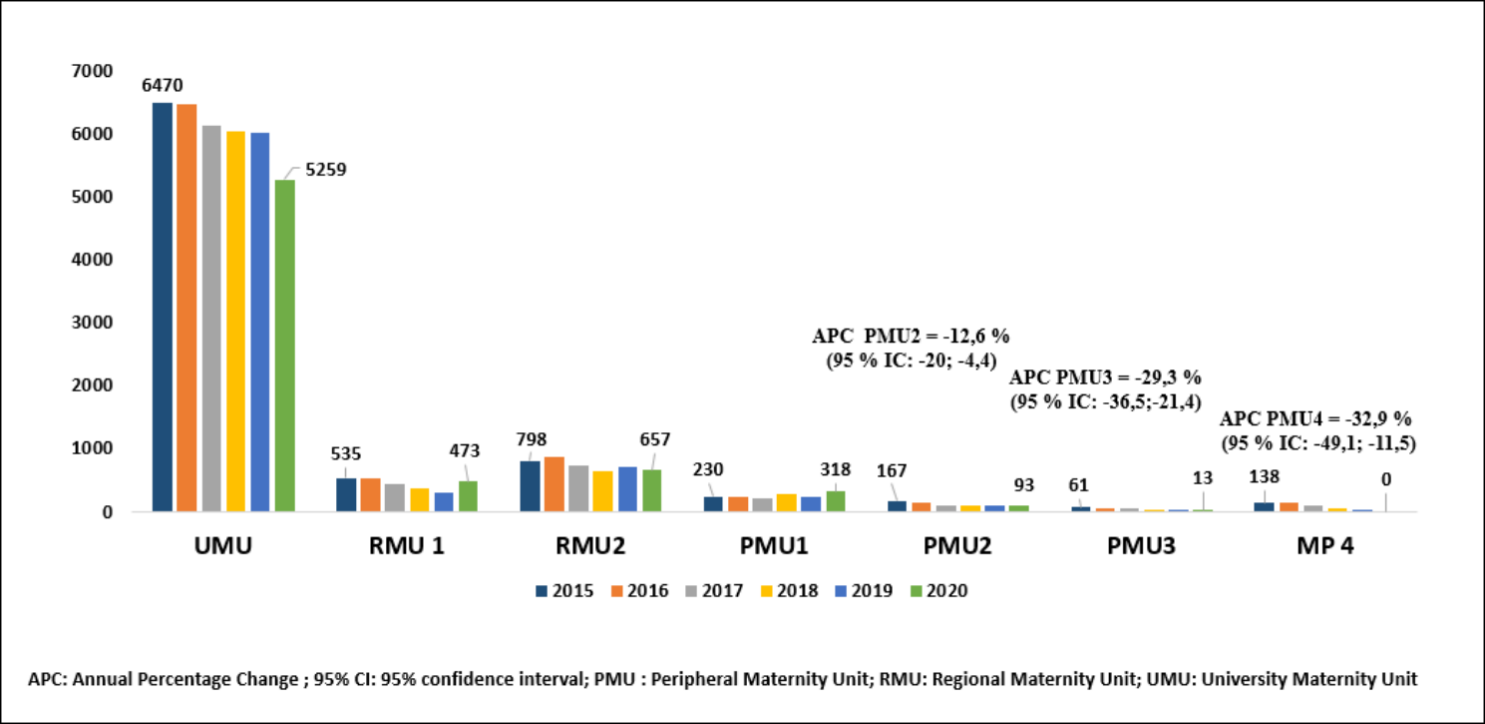
Distribution of the number of deliveries per maternity units during the study period (Monastir, Tunisia). The highest childbirth activities was recorded in the obstetric services with ability to perform emergency caesarean

The number of births in Monastir was 11,520 in 2015 and 11,186 in 2020 (APC= -0.6%; 95%CI: -2.1; 1; p = 0.357). We noted an increase by 1,360 deliveries in private sector (APC = 8.6%; 95%CI: 3; 14.4; p = 0.012), and a decrease by 1,694 deliveries in public sector (APC= -4.3%; 95%CI: -6; -2.4; p = 0.003). The deliveries decrease was significant in UMU, RM2, PM 2, 3 and 4 (Fig. 1). The highest decreases were noted in PMU 2 (APC = -12.6% (95%CI: -20%;-4.4%; p = 0.014), in PMU 3 (APC = -29.3% (95%CI: -36.5%; -21.4%; p = 0.001), and in PMU 4 (APC = -32.9% (95%CI: -49.1%; -11.5%); p = 0.016). The number of maternal deaths was 7 in 2017 and 2 in 2020 (Table 2).

**Table 2:** Description of deliveries in the maternity units and distribution of mean number of deliveries by midwife per year from 2015 to 2020 (Monastir, Tunisia)

	2015	2016	2017	2018	2019	2020	APC (95% CI)	p
**Description of deliveries**								
**MU n (%)**								
**UMU n (%)**	6470 (77.0)	6462 (76.8)	6126 (79.2)	6029 (80.6)	6005 (81.8)	5259 (77.1)	-3.5 (-5.8 ; -1.1)	**0.016**
**RMU 1 n (%)**	535 (6.3)	529 (6.2)	435 (5.6)	375 (5.0)	288 (3.9)	473 (6.9)	-6.2 (-17.3 ; 6.5)	0.235
**RMU 2 n (%)**	798 (9.5)	853 (10.1)	733 (9.4)	646 (8.6)	696 (9.4)	657 (9.6)	-4.8 (-9.1 ; -0.4)	**0.039**
**PMU 1 n (%)**	230 (2.7)	234 (2.7)	210 (2.7)	266 (3.5)	232 (3.1)	318 (4.6)	5.4 (-2.8 ; 14.3)	0.148
**PMU 2 n (%)**	167 (1.9)	141 (1.6)	99 (1.2)	90 (1.2)	90 (1.2)	93 (1.3)	-12.6 (-20 ; -4.4)	**0.014**
**PMU 3 n (%)**	61 (0.7)	51 (0.6)	37 (0.4)	20 (0.2)	13 (0.1)	13 (0.1)	-29.3 (-36.5 ; -21.4)	**0.001**
**PMU 4 n (%)**	138 (1.6)	139 (1.6)	87 (1.1)	49 (0.6)	17 (0.2)	0 (0)	-32.9 (-49.1 ; -11.5)	**0.016**
**Total: N**	8399	8409	7727	7475	7341	6813		
**Distribution of mean number of deliveries by midwife per year**								
**UMU**	108.8	116.9	115.9	120.2	113.2	99.5	-1.3 (-5.8 ; 3.4)	0.473
**RMU 1**	89.1	105.8	108.7	75	57.6	82.6	-6.5 (-18.5 ; 7.3)	0.245
**RMU 2**	94.8	102.2	91.2	79.5	85.1	77.1	-4.8 (-8.8 ; -0.7)	**0.032**
**PMU 1**	46	46.8	42	53.2	46.4	63.6	5.9 (-2.1 ; 14.6)	0.112
**PMU 2**	33.4	28.2	16.5	15	18	18.6	-12.9 (-24.4 ; 0.2)	0.052
**PMU 3**	10.1	10.2	7.4	6.6	6.5	4.3	-14.5 (-20.7 ; -7.9)	**0.004**
**PMU 4**	34.5	34.7	29	16.3	5.6	0	-26.2 (-45.4 ; -1.2)	**0.045**
**Mortality indicators**								
**MD: n**	1	5	7	2	3	2	-13.6 (-49.3 ; 47,5)	0.491
**Maternal M** ^**£**^	11.7	58.3	89.7	26.2	40.0	29.3	-10.8 (-47.1 ; 50.2)	0.574
**Stillbirths: n**	90	108	79	76	85	85	-3.2 (-11 ; 5.2)	0.334
**ND: n**	5	2	5	4	3	NS	-6.6 (-35.5; 35.2)	0.599

APC: Annual Percentage Change; 95% CI: 95% confidence interval; MU: Maternity Unit; PMU: Peripheral Maternity Unit; RMU: Regional Maternity Unit; UMU: University Maternity Unit; MD: Maternal deaths; MM^**£**^: Maternal mortality rate per 100,000 live births; ND: Newborns who died in the delivery room

### Description of deliveries according to human resources

During the study period, the total number of obstetrician-gynecologists performing in public sector increased by five (APC = + 7.8% (CI95%: +4%; +11.7%), p = 0.004) of which two were in RMU. No gynecologist performed in PMUs during the study period. The total number of midwives performing in public sector decreased significantly passing from 115 to 2015 to 90 in 2020 (APC = -5.1% (95%CI: -6.4%; -3.9%); p < 0.001). We noted a decrease by six midwives performing in PMU and RMU (from 34 to 2015 to 28 in 2020) (APC = -3.9% (95%CI: -5%; -2.9%); p < 0.001).

The mean number of deliveries per midwife per year decreased significantly in RMU 2, PMU 3 and 4. The largest decreases were noted in PMU3 (APC = -14.5% (95%CI: -20.7%; -7.9%; p = 0.04) and PMU 4 (APC = -26.2% (95%CI: -45.4%; -1.2%; p = 0.045) (Table 2).

### Indicators of use, availability and accessibility of services

During the study period, the percentages of transfers for delivery were 45.2% for PMU1, 37.4% for PMU3 and 53.4% for PMU4. All transfers were done to UMU (Table 3).

**Table 3:** Transfers for delivery in the maternity units from 2015 to 2020 (Monastir; Tunisia)

MU	Admission for delivery (2015–2020) (n)	Women giving birth (2015–2020) (n)	Transfers for delivery (%)
UMU	36,359	36,350	0.0
RMU 1	3625	2634	27.3
RMU 2	5587	4382	21.6
PMU 1	2716	1489	45.2
PMU 2	807	679	15.9
PMU 3	310	194	37.4
PMU 4	920	429	53.4

MU: Maternity Unit; PMU : Peripheral Maternity Unit; RMU: Regional Maternity Unit; UMU: University Maternity Unit

Indicators of availability of essential obstetric care were satisfied in all Monastir MUs during the 6 years even if PMU 3 and 4 were shut down since 2015. Whereas, if PMU 2 was no longer functioning as a MU, availability of BEOC could not have been satisfied (< 3) (Table 4). PMU 2 and PMU 1 had the closest geographic and economical access to PMU 3 and PMU 4, with travel times under 5 km and costs under 1 TND, respectively (Table 5).

**Table 4:** Evolution of indicators of availability of essential obstetric care from 2015 to 2020 in Monastir (Tunisia)

	All maternity units are functioning		If PMU 3 and 4 were no longer functioning as MUs		If PMU 2 was no longer functioning as a MU	
**Indicators**	Availability of BEOC	Availability of CEOC	Availability of BEOC	Availability of CEOC	Availability of BEOC	Availability of CEOC
**2015**	4.46	2.67	4.46	2.67	**3.57**	2.67
**2016**	4.39	2.63	4.39	2.63	**3.51**	2.63
**2017**	4.34	2.60	4.34	2.60	**3.47**	2.60
**2018**	4.22	2.53	4.22	2.53	**3.38**	2.53
**2019**	4.14	2.48	4.14	2.48	**3.31**	2.48
**2020**	4.15	2.49	4.15	2.49	**3.32**	2.49

MU: Maternity Unit; PMU : Peripheral Maternity Unit; BEOC: basic essential obstetric care facilities per 500,000 population; CEOC: comprehensive essential obstetric care facilities per 500,000 population

**Table 5:** Geographic and economic accessibility between PMU 3 and 4 and other MUs of Monastir (Tunisia)

MU	PMU 3			PMU 4		
	Distance (Km)	Time (min)	Price (TND)	Distance (Km)	Time (min)	Price (TND)
**UMU**	30.2	41	4.429	25.8	34	3.784
**RMU 1**	12.3	23	1.804	22.6	27	3.314
**PMU 1**	25.4	39	3.725	**4.9**	**8**	**0.719**
**RMU 2**	9	16	1.320	22.9	28	3.358
**PMU 2**	**3.7**	**8**	**0.543**	29.3	39	4.297

MU: Maternity Unit; PMU: Peripheral Maternity Unit; RMU: Regional Maternity Unit; UMU: University Maternity Unit; TND: Tunisian Dinar

## Discussion

Our study has highlighted a significant decrease in the number of deliveries in all MU and in the number of midwives in PMU and RMU. A high decrease in the mean number of deliveries by midwife per year was noted in PMU 3 and PMU4. The accessibility and availability indicators remained acceptable even if PMUs 3 and 4 moved from delivery units to perinatal monitoring units.

### Interpretation

In Tunisia, like several other developing nations, many efforts have been made following the implementation of the National Perinatal Program which have led to remedying inequalities in access to maternal and neonatal health services, to ensuring data collection, periodic evaluations and therefore addressing the causes of maternal morbidity and mortality. The number of maternal deaths passed from 7 to 2017 to 2 in 2020. In Tunisia, the number of maternal deaths decreased from 110 to 2000 to 90 in 2017 [15]. According to the guidelines of the National Perinatal Program, a meeting is held including all stakeholders following each maternal death deemed preventable. A search for the possible causes of death and a proposal for new preventive actions is carried out. In our study, the trend of maternal mortality was in line with the international trend. Globally, maternal mortality ratio declined by roughly 38% between 2000 and 2017. 94% of all maternal deaths take place in low and low-middle-income countries [16]. The system for monitoring maternal deaths in public healthcare facilities is based on the partnership and involvement of all levels of the health pyramid, which makes it possible to monitor the trend of maternal mortality [17].

In our study, we found that PMU 2, 3 and 4 had the highest significant decrease in the number of deliveries. It may be explained firstly by the shortage in the number of obstetrician-gynecologists and midwives leading women to prefer to give birth in other MUs offering appropriate medical conditions with sufficient number of health personnel. Moreover, PMUs do not perform deliveries via caesarean section. It seems also possible that this result is due to the decrease in the number of births taking place in the public sector. Indeed, nowadays, Tunisian women prefer to give birth in the private sector since the National Sickness Insurance Fund is taking charge of the deliveries. In Tunisia, the number of Social Security beneficiaries in the public care sector decreased from 2,037,506 in 2008 to 1,818,443 in 2018. In contrast, this number increased in the private care sector from 273,770 to 2008 to 629,250 in 2018. In particular, the number of beneficiaries of deliveries in private sector through the National Sickness Insurance Fund rose from 4,986 to 2007 to 47,637 in 2018 [18]. This information urges global health systems to make it easier to enroll in the social security system. Decision-makers must have dynamic suggestions that are parallel to the social and cultural evolution of communities as a result of the advancement of science and medicine.

Our results in terms of increase in the total number of obstetrician-gynecologists from 2015 to 2020 were concordant with those of the Tunisian health map report. The density of medical specialists in public and private sectors increased from 3 to 10,000 inhabitants in 2016, to 3.2 per 10,000 inhabitants in 2017, and to 3.4 per 10,000 inhabitants 2019 [19]. The decrease in the number of midwives particularly in PMU 2, 3 and 4 can be explained by a shortage in human resources. Indeed, the healthcare personnel is unequally distributed among the 7 public MUs of Monastir. In the UMU, services are ensured by obstetrician-gynecologists and midwives, while in the PMUs, services are provided mainly by midwives. This could explain the growing influx to the UMU. The need for a high-quality technical platform with a minimal risk of complications is growing among women as their education level rises and as society as a whole develops. These factors must be taken into account by international decision-makers in order to identify the true needs of pregnant and provide them delivery services with highly effectiveness. The lack of midwives can be justified by the movement of some ones to the private sector or their migration abroad, due to their perception that their working conditions are not optimal. Furthermore, there is a deficiency in the recruitment of health care professionals. Another possible explanation is the decrease in the number of women giving birth and the use of social media for the choice of maternity according to the published statutes between pregnant women groups [20].

WHO discussion about the viability of peripheral maternities in developing countries should be based on delivery service use and the transfer rate. Since gynecologists are in limited supply in rural regions, family doctors who are skilled in birthing and caesarean sections can help fill the job. Based on our findings highlighting the highest significant decrease in the number of deliveries in PMU 2, 3 and 4, it appeared that PMUs 2, 3 and 4 can no longer function as a MU. When considering the number of deliveries per midwife per year, its trend was stable in PMU 2, while there was a significant decrease in PMU 3 and 4. Moreover, even though PMU 3 and 4 are no longer functioning as delivery units, both indicators of “Availability of BEOC” and “Availability of CEOC” meet the recommended minimum acceptable level (more than 4 BOEC care facilities per 500,000 population and more than 1 COEC facilities per 500,000 population). Consequently, PMU 3 and 4 can perform other components of the National Perinatal Program such as the promotion of breastfeeding, family planning, screening for female cancers and no longer perform deliveries. According to a study conducted in seven rural counties and villages in Shanxi Province (China), the number of CEOC facilities was appropriate in all countries. Only four countries met the recommended level of BEOC facilities [21]. In order to ensure that a facility is fully functioning as a BEOC or CEOC, the whole health system must be functioning. This should include facility infrastructure, human resources, health care financing, maternal health policy, drugs and supplies, acquisition and distribution of equipment, the health information system and referral systems [22]. In the area of globalization, the speed of circulation of information, the supply of care must take into consideration the preferences of the population.

### Limitations and strengths

Our study findings were subject to two limitations. First, it did not analyze the economic cost of some PMUs with low numbers of women giving birth. Secondly, the quality indicators used should be adapted to the governorate studied with region-specific minimum recommended levels. Actually, global indicator lists are not necessarily appropriate for districts [3]. The strength of this work was the fact that it is one of the few studies that have evaluated the activity of MUs in Tunisia, comparing all the MUs in the same governorate.

### Recommendations

We recommend to evaluate all the components of the National Perinatal Program taking into account the rapid changes of the countries economies. Obstetric services without ability to perform emergency caesarean section should not exist in current practice. We propose for developing countries, the association of conditions for replacing the MUs activities (Appendix B). The next stage of research is to validate our recommendations on all maternity units.

## Conclusion

In our study, we have highlighted the important role of MUs in Monastir and described the state of progress of the 3rd component of the National Perinatal Program: the childbirth in an assisted environment. We have also shown the interest of the continuous evaluation of MUs activities. Indeed, for better management of human resources and to meet the expectations of pregnant women in the area of globalization, efforts must focus on improving the quality of care with better availability of medical assistance.

### Electronic supplementary material

Below is the link to the electronic supplementary material.

Supplementary Material 1

## Data Availability

The datasets used and/or analyzed during the current study are available from the corresponding author on reasonable request.

## References

[1] Reproductive health indicators (2006). : guidelines for their generation, interpretation and analysis for global monitoring.

[2] Guidelines for monitoring the availability and use of obstetric services. : revised manual Aout 1997. Columbia University: UNICEF. Center for Population and Family Health (https://www.publichealth.columbia.edu/sites/default/files/pdf/unguidelinesen.pdf, accessed 14 May 2022).

[3] Maternal mortality. World Health Organization. 22 February 2023 https://www.who.int/news-room/fact-sheets/detail/maternal-mortality.

[4] Selecting indicators of (1998). Reproductive health: a guide for district managers, field-testing version.

[5] Santana IR, Mason A, Gutacker N, Kasteridis P, Santos R, Rice N. Need, demand, supply in health care: working definitions, and their implications for defining access. Health Econ Policy Law. 2021;1–13. 10.1017/S1744133121000293 36515132

[6] Governorate of Monastir. Tunisian Republic: Ministry of Industry, Energy and Mines (http://www.tunisieindustrie.nat.tn/fr/doc.asp?docid=600&mcat=13&mrub=105, accessed 13 February 2022).

[7] Statistics Tunisia. : National Institute of Statistics (http://www.ins.tn/statistiques/111, accessed 13 February 2022).

[8] Google Maps. (https://www.google.com/maps/dir///@36.8279552,10.2039552,12z?hl=fr, accessed 12 February 2022).

[9] 1 TND to EUR exchange. rate - How much is Tunisian Dinar in Euro? moneyexchangerate.org. (https://moneyexchangerate.org/currencyexchange/tnd/eur/1, accessed 21 October 2022).

[10] World Health Organization, editor (2006). Reproductive health indicators: guidelines for their generation, interpretation and analysis for global monitoring.

[11] World Health Organization, United Nations Population Fund, Mailman School of Public Health. Averting Maternal Death and Disability, Monitoring emergency obstetric care: a handbook. Surveillance des soins obstétricaux d’urgence : manuel d’utilisation 2009; 152.

[12] Biswas AB, Das DK, Misra R (2005). Availability and use of emergency obstetric care services in four districts of West Bengal, India. J Health Popul Nutr.

[13] What is Stillbirth?. : Centers for Disease Control and Prevention. (https://www.cdc.gov/ncbddd/stillbirth/facts.html, accessed 11 February 2022).

[14] Tunisia Gasoline prices (https://fr.globalpetrolprices.com/Tunisia/gasoline_prices/, accessed 17 January 2022).

[15] Maternal Mortality. : University of Oxford. Our World in Data (https://ourworldindata.org/maternal-mortality, accessed 14 May 2022).

[16] Maternal mortality. Geneva: World Health Organization. ; 2019 (https://www.who.int/news-room/fact-sheets/detail/maternal-mortality, accessed 14 May 2022).

[17] Chelli D, Dimassi K, Zouaoui B, Sfar E, Chelli H, Chennoufi MB (2009). Evolution of maternal mortality in a level 3 tunisian maternity from 1998 to 2007. J Gynecol Obstet Biol Reprod.

[18] Statistics. National Health Insurance Fund of Tunisia (http://www.cnam.nat.tn/stat.jsp, accessed 7 February 2022).

[19] Smith R, Alvarez C, Crixell S, Lane MA. The Food, Feelings, and Family Study: comparison of the efficacy of traditional methods, social media, and broadcast email to recruit pregnant women to an observational, longitudinal nutrition study. BMC Pregnancy Childbirth. 2021 Mar 12;21(1):203. 10.1186/s12884-021-03680-1 PMC7953646 33711946

[20] Health map. : National health portal in Tunisia (http://www.santetunisie.rns.tn/fr/sante-en-tunisie/la-sante-scolaire-et-universitaire/carte-sanitaire, accessed 17 January 2022).

[21] Gao Y, Barclay L (2010). Availability and quality of emergency obstetric care in Shanxi Province, China. Int J Gynaecol Obstet.

[22] Kongnyuy E, Hofman J, van den Broek N (2009). Ensuring effective essential Obstetric Care in resource poor settings. BJOG.

