# Supplementary material for: Quality indicators of public maternity units in the governorate of Monastir (Tunisia)
Source: BMC Pregnancy Childbirth. 2023 Oct 16;23:731. doi: 10.1186/s12884-023-05781-5 (PMC10577896; doi:10.1186/s12884-023-05781-5)
Supplement: Supplementary file 1 — Supplementary Material 1 [file 12884_2023_5781_MOESM1_ESM.docx]

**Appendix A: Indicators calculated to describe the activity of MUs**

| **Indicator** | **Numerator** | **Denominator** |
| --- | --- | --- |
| **Maternal mortality rate** (per 100000 live births) | the number of maternal deaths *100000 | total number of live births |
| **Rate of stillbirths** (per 1000 live births) | number of stillbirths*1000 | total number of live births |
| **Rate of new-borns who died in the delivery room** (per 1000 live births) | number of new-borns who died in the delivery room *1000 | total number of live births |
| **Number of deliveries per midwife per year** | annual number of deliveries accomplished by midwives | number of midwives |
| **Percentage of women giving birth among admission** | number of women giving birth *100 | total number of admissions for delivery |
| **Availability of BEOC** (per 500000 population) | number of facilities with functioning basic care *500000 | total population |
| **Availability of CEOC** (per 500000 population) | number of facilities with functioning comprehensive care* 500 000 | total population |
| **Percentage of transfers after delivery** | annual number of transfers after delivery *100 | number of deliveries performed in the same period |
| **Price of each trip between maternity units** [11] | number of kilometers * price of the gasoline per liter * consumption of the vehicle per kilometer (average passenger vehicle consumes 0.07 liters per km) |  |

BEOC: basic essential obstetric care

CEOC: comprehensive essential obstetric care

**Appendix B: recommendations:** conditions for replacing the MUs activities

| ***Indicator*** | ***Criteria*** | ***Standard*** |
| --- | --- | --- |
| Geographic accessibility | Perimeter around the MU and the target population | Less than 20 km in condition of availability of transportation |
| Delivery services use | Births by midwife by year | Less than a mean of 50 in consecutive two years |
| Transfer rate | Rate of transfers for delivery by Women giving birth in MU | More than 30% |
| Disponibility of Skilled doctors with ability to perform emergency caesarean | Skilled: must have performed more than 50 assisted caesarean sections | Less than 4 skilled doctors |
| Availability of an operating room | Number of operating room in MU | Less than one |
| Disponibility of anesthesia technicians | Number of anesthesia technicians by operating room | Less than five by operating room |
| Mother satisfaction | Rate women giving birth satisfied | Less than 80% |
